# Identification of a Potential Target of Capsaicin by Computational Target Fishing

**DOI:** 10.1155/2015/983951

**Published:** 2015-12-03

**Authors:** Xuan-yi Ye, Qing-zhi Ling, Shao-jun Chen

**Affiliations:** ^1^College of Ecology, Lishui University, Lishui, Zhejiang 323000, China; ^2^Department of Traditional Chinese Medicine, Zhejiang Pharmaceutical College, Ningbo 315100, China

## Abstract

Capsaicin, the component responsible for the pungency of chili peppers, shows beneficial effects in many diseases, although the underlying mechanisms remain unclear. In the present study, the potential targets of capsaicin were predicted using PharmMapper and confirmed via chemical-protein interactome (CPI) and molecular docking. Carbonic anhydrase 2 was identified as the main disease-related target, with the pharmacophore model matching well with the molecular features of capsaicin. The relation was confirmed by CPI and molecular docking and supported by previous research showing that capsaicin is a potent inhibitor of carbonic anhydrase isoenzymes. The present study provides a basis for understanding the mechanisms of action of capsaicin or those of other natural compounds.

## 1. Introduction

**Figure 1:**
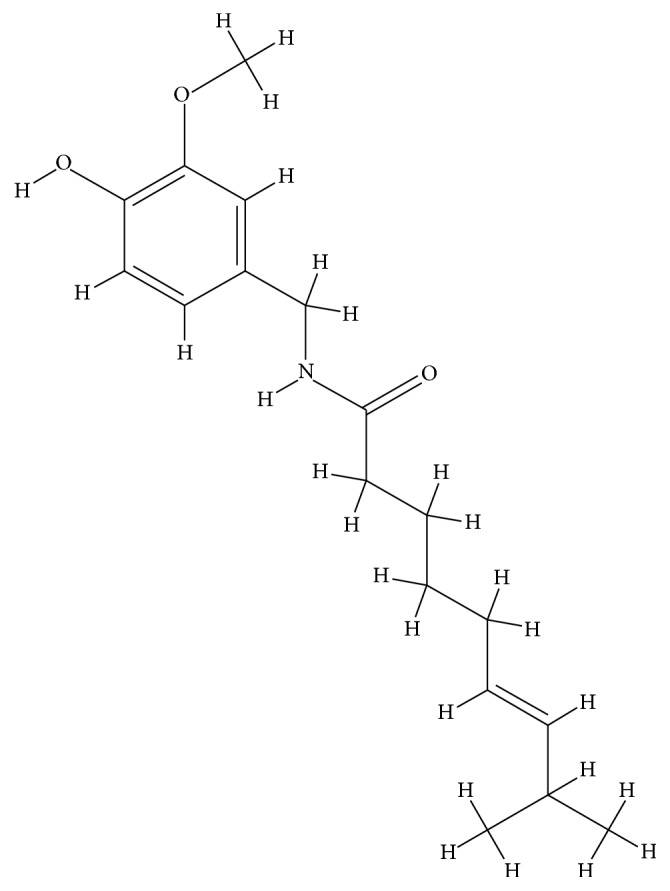
The chemical structure of capsaicin (PubChem CID: 1548943).

Capsaicin (Figure 1), the component responsible for the pungency of chili peppers, is an alkaloid from the* Capsicum *species, which is used worldwide in foods, spices, and medicines [1–4]. Capsaicin has been used as traditional medicine to treat muscular pain and headaches, to improve circulation, for its gastrointestinal protective effects, and to fight against many types of cancer [4, 5]. It is commonly added to herbal formulations because it acts as a catalyst for other herbs and aids in their absorption [4]. As a result, capsaicin has become an exciting pharmacological agent and its utility in different clinical conditions is being explored [1]. However, the mechanisms underlying the therapeutic effects of capsaicin remain unclear [1].

Target fishing, or target identification, is an important step in modern drug development that explores the mechanism of action of bioactive small molecules by identifying their interacting proteins [6, 7]. In recent years, a large number of computational target fishing methods have been developed [8]. For example, reverse or inverse docking represents a useful tool that involves docking a small-molecule drug/ligand into the potential binding cavities of a set of clinically relevant macromolecular targets [9]. Identification of the top-ranking targets based on their binding affinity with the drug/ligand may be relevant for drug repositioning and/or rescue [9]. In recent work from our group, computational tools were used to identify targets of Danshensu and Tanshinone IIA [10, 11]. Computational target fishing technologies have increased our ability to efficiently and effectively screen for targets in a high-throughput format, which is expected to have a large impact on drug development [6, 8].

In the present study, potential targets of capsaicin were predicted by reverse docking and confirmed via chemical-protein interactome (CPI) and molecular docking. The present study describes a computational drug repositioning method and explores its potential for elucidating the mechanism of action of natural compounds.

## 2. Methods

### 2.1. Targets Predicted by PharmMapper

PharmMapper is a web server for potential drug target identification based on the use of a pharmacophore mapping approach [12]. It automatically finds the best mapping poses of the query molecule against all the pharmacophore models in PharmTargetDB and lists the top N best-fitted hits with appropriate target annotations, as well as the aligned poses of the respective molecules [12].

The molecular file of capsaicin was downloaded from the PubChem database (CID: 1548943) and uploaded to the PharmMapper server. The search started using the maximum generated conformations at 300 by selecting “all targets (7302)” option and default value of 300 for the number of reserved matched targets as described previously [10, 11, 13]. The default settings were used for other parameters.

### 2.2. Targets Checked by the CPI

The CPI refers to the interaction information of a panel of chemicals across a panel of target proteins in terms of binding strength and binding conformation for each chemical-protein pocket pair [14]. Both DRAR-CPI and DDI-CPI are the servers for computational drug repositioning via the CPI [15, 16].

The molecular file of capsaicin was downloaded and pretreated following the web instructions as described previously [11, 15]. Then, it was submitted to the DRAR-CPI and DDI-CPI servers. Parameters were set to default values.

### 2.3. Molecular Docking

Molecular docking is a computational procedure that attempts to predict noncovalent binding of macromolecules or a macromolecule (receptor) and a small molecule (ligand) efficiently [17]. Autodock Vina in PyRx 0.8 is a new program for molecular docking and virtual screening that has been widely used [17–19].

The target protein was prepared using the protein preparing tool in TCM Database@Taiwan (http://dock.cmu.edu.tw/ligand.php), which can extract ligands from binding sites, protonate protein structures, and show ligand coordinates and radius information as described previously [10, 11]. Then, the ligand capsaicin was pretreated through OpenBabel in PyRx 0.8. During the docking procedure, the grid box was centered to cover the binding site residues and to allow the ligand to move freely [10, 11]. The box was set to 10 × 10 × 10 nm, and the center coordinates are shown in Table 2. Other parameters were set to default values.

### 2.4. Visualization

The 3D visualizations of the complex structure were performed using soft PyMol, and the diagrams of chemical-protein interactions were prepared using Ligplot software.

## 3. Results

### 3.1. Target Prediction by PharmMapper

**Figure 2:**
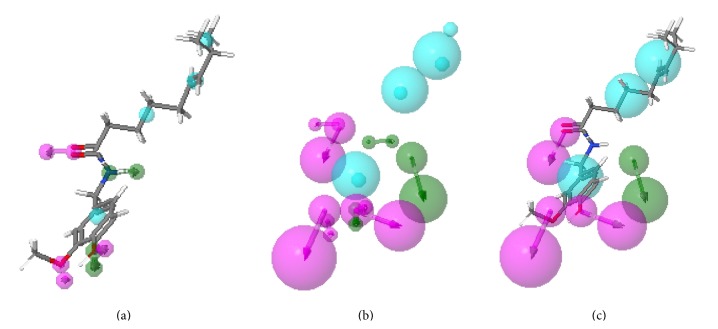
Alignment of capsaicin and pharmacophore model of CA2. (a) Capsaicin features. (b) Pharmacophore model of CA2. (c) Molecular and pharmacophore model. Note: pharmacophore features are indicated by color as follows: hydrophobic, cyan; positive, blue; negative, red; donor, green; and acceptor, magenta.

Ranking by fit score in descending order and the top ten disease-related targets are shown in Table 1. Carbonic anhydrase 2 (CA2) (PDB ID: 1BNV, 1I9Q, and 1I9O) ranked number one, three, and nine respectively. The pharmacophore model (1BNV) shows three hydrophobic sites, one donor, and three acceptors (Figure 2). Moreover, the pharmacophore model showed that CA2 is well matched with capsaicin (Figure 2). These results indicate that CA2 may be a potential target of capsaicin. Therefore, CA2 was selected for further investigation.

### 3.2. Targets Verified by Chemical-Protein Interactome

When a drug is uploaded to the DRAR-CPI server, it is “hybridized” with all targets using the DOCK program [15]. Table 2 shows the results of DRAR-CPI for capsaicin-CA (12 and 3), including the docking score and *Z*′-score.

**Figure 3:**
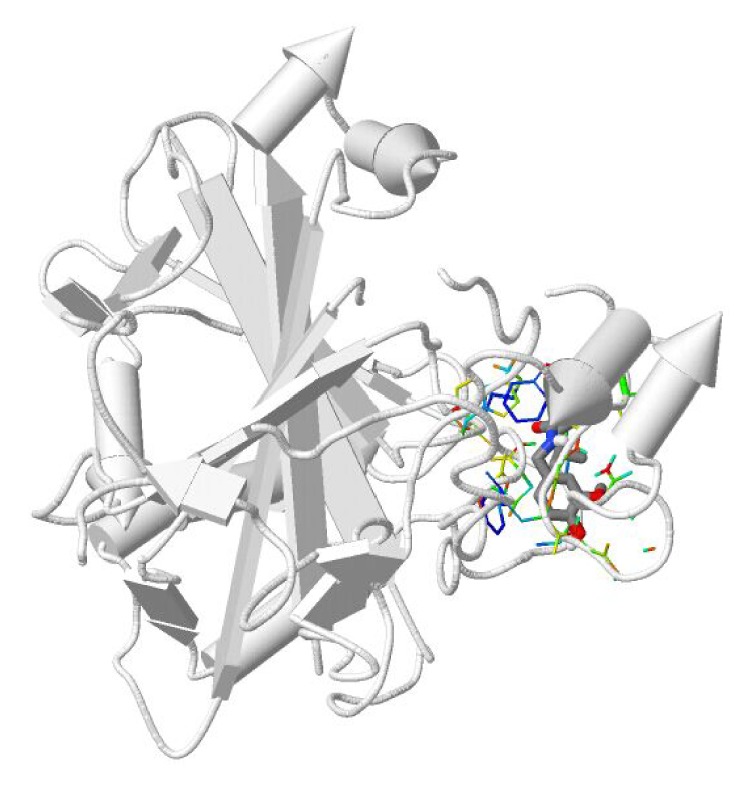
Visualization of a capsaicin-CA2 complex captured from the DDI-CPI server. Note: protein chain: rocket; drug: stick; key residues: colorful line.

When a molecule is submitted to DDI-CPI, the server will dock it across 611 human proteins, generating a CPI profile that can be used as a feature vector of the preconstructed prediction model [16]. As shown in Table 2, four CA isoforms, including CA1, 2, 4, and 13, docked with capsaicin. The docking score of capsaicin-CA2 was −5.9 kcal/mol. Furthermore, the binding pattern of capsaicin-CA2 complex can be visualized in Figure 3.

### 3.3. Molecular Docking

**Figure 4:**
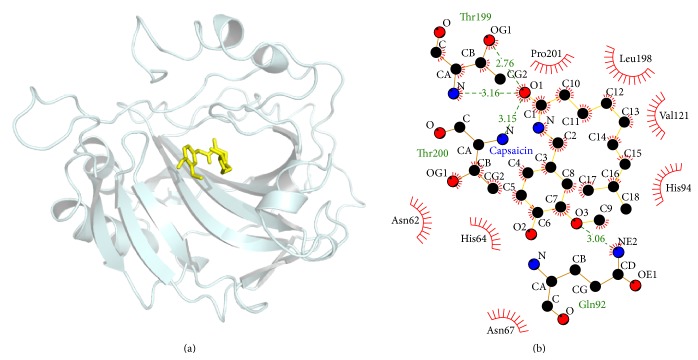
Molecular interactions between capsaicin and CA2. (a) 3D structure of the CA2 (1BVN)-capsaicin complex by PyMol. Capsaicin: yellow; hydrogen bond: red dash line. (b) 2D interaction scheme by Ligplot. Capsaicin: yellow; C, N, and O atoms are represented in black, blue, and red; hydrophobic contacts are presented in brick red.

Upon docking using Autodock Vina in PyRx 0.8, the lowest binding energy of the capsaicin-CA2 complex was −6.2 kcal/mol (Table 3). As shown in Figure 4, the ligand capsaicin formed four hydrogen bonds with the active site residues (Gln92, Thr199, and Thr200). A number of hydrophobic interactions are depicted in Figure 4(b). Many residues, including Asn62, His64, Asn67, His94, Val121, Leu198, and Pro201, formed hydrophobic contacts with capsaicin.

## 4. Discussion

The identification of drug targets in the human genome is important for the development of new pharmaceutical products and the allocation of resources in academic and industrial biomedical research [20]. Various innovative computational tools have been developed to integrate biological data such as regulatory networks, molecular pathways, and cell phenotypes, which facilitates the interpretation and prediction of the biological activities of drugs and their targets [8, 21]. Reverse or inverse docking is a powerful tool for drug repositioning and drug rescue [9]. Recently, PharmMapper, a reverse docking server, was used to identify potential targets of small molecules derived from* Indigofera* species [22] and for the computational prediction of breast cancer targets for 6-methyl-1,3,8-trichlorodibenzofuran [13]. In our previous reports, we used the PharmMapper server to identify potential targets of active compounds from Danshen, a traditional Chinese medicine [10, 11]. We therefore used PharmMapper, a powerful computational tool, to identify CA2 as the main disease-related target of capsaicin in the present study (Table 1). The CA2 pharmacophore confirmed the alignment of molecular features with capsaicin (Figure 2).

The use of CPI together with systems biology-based integrative computational strategies is an essential complement, if not an alternative, to current drug evaluation methods [14]. In a DRAR-CPI job, potential drug targets with *Z*′-score <−1 are considered as the favorable targets and those with *Z*′-score >1 are considered as the unfavorable targets [15]. In a DDI-CPI job, the docking scores for each drug in the training set are generated against the 611 library targets [16]. In the present study, *Z*′-score in DRAR-CPI and docking score in DDI-CPI indicated that CA2 is a target of capsaicin and should be further investigated. These results were consistent with the reverse docking results (Table 1).

Understanding the interactions between proteins and biologically relevant ligands is an important step towards identifying the functions of proteins [23]. The hydrophobic surface of the active site cavity of CA2 contains the residues Ala121 and 135; Val207; Phe91; Leu131, 138, 146, and 109; and Pro201 and 202; and the hydrophilic surface consists of His64, 67, and 200; Asn69; Gln92; Thr199; Tyr7; and Val62 [24]. Thr199 plays a significant role by forming two hydrogen bonds with the carboxyl group of Glu106 and zinc hydroxide [24]. Residues Asn67 and Leu198 protrude towards the Zn^2+^ ion and reduce the volume of the active site cavity considerably [24]. His64, Asn67, and Gln92 residues are involved in histidine recognition [24]. In short, these residues play key roles in ligand-protein interactions. The original ligand sulfonamide forms hydrogen bonds with residues Gln92, His119, Thr199, and Thr200 and forms hydrophobic interactions with Phe131 [25]. Figure 4(b) shows that capsaicin can form hydrogen bonds with Gln92, Thr199, and Thr200 and has hydrophobic interactions with Asn62, His64, Asn67, His94, Val121, Leu198, and Pro201. The structural details indicate that capsaicin may interact with CA2 via these key residues [24]. A previous study reported that capsaicin has *K*_*i*_ of 696.15 *μ*M against hCA I and of 208.37 *μ*M against hCA II, showing unique inhibition profiles against both CA isoforms I and II and suggesting that capsaicin is a selective inhibitor of both cytosolic CA isoenzymes [26].

CAs, a group of ubiquitously expressed metalloenzymes, are involved in numerous physiological and pathological processes, including gluconeogenesis, lipogenesis, ureagenesis, tumorigenicity, and the growth and virulence of various pathogens [27]. In addition to the established role of CA inhibitors (CAIs) as diuretics and antiglaucoma drugs, the potential of CAIs as novel antiobesity, anticancer, anti-infective, and anti-Alzheimer's drugs was recently shown [27]. Taken together with previous results, our findings suggest that capsaicin may play a role in these diseases through its effect on CA2.

In the present study, potential targets of capsaicin were identified using PharmMapper and confirmed via CPI and Autodock Vina. Our results identified CA2 as a potential target of capsaicin, although further studies are necessary to determine their precise interaction. The present study demonstrated that computational drug repositioning is a useful strategy to screen for targets of capsaicin or other natural compounds and suggested a mechanism of action of capsaicin.

## Figures and Tables

**Table 1:** Top ten potential disease-related targets of capsaicin predicted by PharmMapper.

Rank	PDB ID	Name	Fit score	Disease
1	1BNV	Carbonic anhydrase 2	4.856	Autosomal recessive osteopetrosis type 3
2	1IZ2	Alpha-1-antitrypsin	4.727	Chronic obstructive pulmonary disease
3	1I9Q	Carbonic anhydrase 2	4.581	Autosomal recessive osteopetrosis type 3
4	5P21	GTPase HRas	4.447	Costello syndrome, cancer
5	1B0F	Leukocyte elastase	4.301	Cyclic hematopoiesis
6	2DUX	Aldose reductase	4.228	Diabetes, galactosemia
7	3BYS	Protooncogene tyrosine-protein kinase LCK	4.225	Leukemias
8	1RLB	Transthyretin	4.028	Amyloidosis
9	1I9O	Carbonic anhydrase 2	4.002	Autosomal recessive osteopetrosis type 3
10	1R1H	Neprilysin	3.982	Acute lymphocytic leukemia

**Table 2:** Results of capsaicin-CA interactome by DRAR-CPI and DDI-CPI.

DRAR-CPI				DDI-CPI		
PDB ID	Name	Docking score	*Z*′-score	PDB ID	Name	Docking score
1JD0	CA 12	−45.9539	1.48099	3CZV 2FOY	CA 13 CA 1	−6.4 −6.1
1Z93	CA 3	−41.1238	1.79532	2FOU 3FW3	CA 2 CA 4	−5.9 −5.7

**Table 3:** The center coordinates of the binding site and the lowest binding energy by molecular docking.

PDB ID	Name	Center (*x* × *y* × *z*)	Binding affinity
1BNV	Carbonic anhydrase 2	−4.03 × 4.83 × 14.43	−6.2 kcal/mol
